# Online detection of error-related potentials boosts the performance of mental typewriters

**DOI:** 10.1186/1471-2202-13-19

**Published:** 2012-02-15

**Authors:** Nico M Schmidt, Benjamin Blankertz, Matthias S Treder

**Affiliations:** 1 Machine Learning Laboratory, Berlin Institute of Technology, Berlin, Germany; 2 Artificial Intelligence Laboratory, Department of Informatics, University of Zurich, Switzerland, Andreasstrasse 15, 8050 Zurich, Switzerland

**Keywords:** Brain-computer interface, Electroencephalography, ERP-Speller, Error-related potentials, Information transfer rate

## Abstract

**Background:**

Increasing the communication speed of brain-computer interfaces (BCIs) is a major aim of current BCI-research. The idea to automatically detect error-related potentials (ErrPs) in order to veto erroneous decisions of a BCI has been existing for more than one decade, but this approach was so far little investigated in online mode.

**Methods:**

In our study with eleven participants, an ErrP detection mechanism was implemented in an electroencephalography (EEG) based gaze-independent visual speller.

**Results:**

Single-trial ErrPs were detected with a mean accuracy of 89.1% (AUC 0.90). The spelling speed was increased on average by 49.0% using ErrP detection. The improvement in spelling speed due to error detection was largest for participants with low spelling accuracy.

**Conclusion:**

The performance of BCIs can be increased by using an automatic error detection mechanism. The benefit for patients with motor disorders is potentially high since they often have rather low spelling accuracies compared to healthy people.

## Background

Brain-computer interfaces (BCIs) establish a direct communication link between the human brain and an electronic device [1,2]. The intent of the user is 'decoded' from her/his brain signals, *e.g*. from electroencephalography (EEG) or magnetoencephalography (MEG), and transformed into control commands for an external device. A great amount of research focuses on restoring sensory-motor functionality or communication ability in people who suffer from motor disorders, such as amyotrophic lateral sclerosis (ALS) [3]. For ALS patients, BCI is a promising technology [4], because it can restore their ability to communicate wishes and needs and to interact with their environment, *e.g*. by controlling a spelling application [5,6], a PC-cursor [7], or a wheelchair [8].

In EEG-based BCIs, many approaches capitalize on event-related potentials (ERPs) that arise as a response to sensory stimulation. An often targeted ERP component is the P300, a positive deflection at central and parietal electrode sites about 300 ms after the presentation of a stimulus that the user is attending to. The P300 and other ERP components have been successfully used as features in BCI spelling applications in order to identify the characters the user intends to write. The classic spelling application is the so-called P300-speller introduced by Farwell and Donchin [9], which is denoted here more specifically as Matrix Speller. It consists of a 6 × 6 matrix of characters. Each row and column is intensified (flashed) briefly in a random order, while the user is directing her/his gaze to the target character. Since detecting the P300 in single trials is intricate, the intensification sequence is repeated several times. By optimizing the number of sequence repetitions, the duration of the flashes, as well as the classification methods, a spelling speed of up to 5.8 characters per minute has been reported [10].

Compared to alternative technologies such as eye-trackers or EOG-based systems, where users communicate with up to 10 words per minute [11], this spelling speed is rather low. Therefore, currently, the clinical application of BCI spellers is mainly of interest in cases of severe oculomotor impairment. It has been shown however, that the spelling accuracy of the Matrix Speller also depends on the user's capability to direct her/his eye gaze to the desired target character. The accuracy drops critically low when the user is required to fixate a dot in the center of the matrix with her/his eyes [12,13].

Recently, some novel approaches for visual spellers have been proposed to overcome this restriction [14-16]. Our study builds on the so-called Center Speller [15,17], but the method could similarly be applied to other spellers. The Center Speller is a visual ERP-speller, which uses a two-step selection process: first, six groups of five characters are presented one by one in a fast sequence in the center of the screen. The user is attending to the target group, *i.e*. is waiting for its appearance. In the second step, the characters of the previously selected group are presented in the same way. In both steps, the six choices are coupled to simple geometric shapes of unique colors in order to facilitate the allocation of attention in fast stimulus sequences (see [15] and method section for a more detailed description).

As mentioned above, a bottleneck of current state-of-the-art BCIs is the low information throughput. For the Center Speller, a previous study showed an average spelling speed of about 1.5 characters/minute at 10 sequence repetitions (*i.e*. each of the six groups/characters is presented 10 times) [15]. Several approaches have been explored in order to increase communication speed. One possibility is to reduce the number of repetitions, at the risk of decreasing spelling accuracy and fatigue of the participant. An optimal balance between the number of repetitions and accuracy can be achieved by means of a dynamic stopping method that statistically evaluates the confidence of the classification after each intensification sequence. If the classifier is confident about the selection, the presentation sequence is stopped [18-20]. Another factor affecting communication speed is experimental overhead. In the Center Speller, the selection process for each character begins with a countdown before the sequence presentation starts. Furthermore, it contains a few animations and presentation of the selected character (feedback). Spelling speed can be increased by reducing the durations of countdown, feedback and animations. As with reduction of repetitions, a potential drawback in reducing the overhead is that a too-short spelling process could be exhausting to the user because it may require more attention.

A different to increasing the spelling speed is the detection of error-related potentials (ErrPs). ErrPs are a certain type of ERPs that are present in the EEG signals when the user is aware of erroneous behavior. ErrPs probably arise in the anterior cingulate cortex, a brain area involved in processing of emotion and attention, and are thus found over central and prefrontal electrode positions [21]. They are characterized by an early negative voltage deflection over fronto-central regions, referred to as error-negativity (*N*_E_) or error-related negativity, followed by a positive deflection over parietal regions, referred to as error-positivity (*P_E_*) [22]. The characteristics of the ErrPs vary, depending on the situation in which the erroneous behavior was perceived. In errors during a choice reaction task, where the subjects respond to a stimulus by pressing a button, erroneous button presses yield ErrPs that are sometimes referred to as "response ErrPs". The *N_E _*appears after 80 ms, the larger *P_E _*follows around 200-500 ms relative to the button press [23,24]. When users perform wrong in a reinforcement learning task and receive a feedback indicating the wrong action, the observed main component is the *N_E _*around 250 ms after the stimulus and this is referred to as "feedback ErrP" [21]. When users observe erroneous behavior of other persons, the so-called "observation ErrP" appears to be similar to the feedback ErrP [25]. In BCI experiments the situation is different. Errors are usually neither caused by the user's action nor by another person the user is observing but by the misclassification of the BCI. Interestingly, in this case ErrPs also arise, with an *N_E _*component after 270 ms and a larger *P_E _*component 350-450 ms after the appearance of the BCI's feedback [26-31]. Ferrez and Millán [26] coined the term "interaction ErrP" for this type of ErrP.

Few studies have been conducted so far on the detection of interaction ErrPs. ErrP detection has been used to detect error trials offline in EEG-data of motor imagery experiments [32], in EEG-data of button press experiments with artificially induced errors [28], in MEG-data of covert attention experiments [33], as well as in EEG-data of Matrix Speller experiments [31]. Dal Seno *et. al *[34] used online ErrP detection in pseudo-online Matrix Speller experiments with five healthy participants, and later in online Matrix Speller experiments with three participants [30]. Spüler [29] showed successful online ErrP detection with the Matrix Speller in 12 healthy participants (29.5% increase of bit rate) and 4 patients with motor disorders (35.6% increase of bit rate).

The aim of the present study was to investigate, whether the communication rate of gaze-independent BCIs can be increased using online detection of ErrPs. To this end, an error detection mechanism was implemented in the Center Speller. If an error potential was detected by the ErrP classifier upon presentation of the classified symbol, the selection was vetoed and the trial was restarted. The communication rate in characters/minute of this modified speller was then compared to the communication rate of the Center Speller without error detection. Moreover, two different ErrP classifiers were compared; one classifier was trained on Center Speller data and another one was trained on data of a calibration experiment and was then applied in the Center Speller experiment. In the Methods section, both classifiers are introduced and the experimental protocol is explicated. In the Results section, we report on the neurophysiological data and on the impact of error potential detection on communication rate.

## Methods

### Center speller

**Figure 1:**
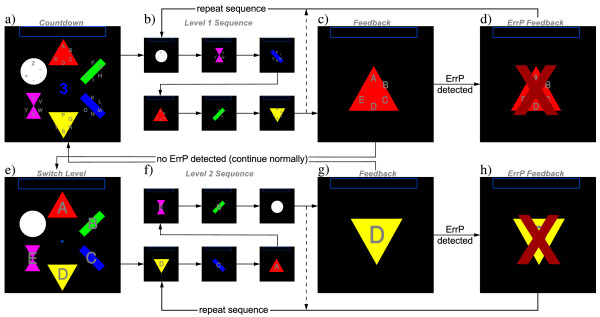
**Program flow of the Center Speller with ErrP detection**. Spelling starts with a countdown (a), followed by the stimulation sequence (b), wherein each symbol is presented sequentially in a randomized order. Then, the feedback (selected symbol group) is presented to the user (c), and the ErrP classifier evaluates the brain response (ErrP present/absent). If an ErrP is detected, a red 'X' indicates the error (d) and the stimulus sequence repeats. Otherwise, the algorithm proceeds with level 2 (e) wherein the selection process is repeated on the single character level (f-h). Finally, the selected character is appended to the phrase shown at the top of the screen and the next trial is started.

The selection process in the Center Speller (see Figure 1) is split into two levels: Thirty characters are divided into six groups of five characters each. In the first level, the six groups (ABCDE, FGHIJ, KLMNO, PQRST, UVWXY and Z_., <) are presented several times one by one in a random order for 100 ms with 100 ms inter-stimulus interval (total stimulus onset asynchrony is 200 ms). The group containing the target character has to be selected by attending to it. In the second step, the single characters from the previously selected group are presented in the same way. Since six selectable stimuli are presented in a random order with equal frequency, the presentation of the target stimulus constitutes a rare event and is supposed to modulate the ERP. Each group (in level one) and each character (in level two) is placed on a polygon with an unique geometric shape and color (red triangle, green bar, blue bar, yellow downward triangle, magenta hourglass and white circle). This way, the two visual features, color and form, are additionally provided by the stimuli.

After having presented all stimulus sequences of a level, the feedback indicating the selected group or character is presented for 1s. In case of a wrong selection by the classifier, an ErrP is elicited and can be detected by another classifier. If this classifier detects an ErrP, a red 'X' appears over the feedback to indicate that the selection by the group or character is vetoed and the stimulus sequence is immediately repeated.

For the case that a wrong group was selected but no ErrP could be detected, the character level provides a backdoor indicated by an accent character". By selecting the backdoor, one returns to the group level without spelling a character. If a wrong selection occurred at the character level, a correction can be made via the less-than symbol ' < ', which serves as a backspace.

### ErrP calibration speller

In order to train an ErrP classifier, a sufficient number of trials needs to be collected. However, the Center Speller yields a spelling speed of about two characters per minute, *i.e*. four samples of feedback evaluation per minute. In other words, to obtain a moderate training set for the ErrP detection, participants need to engage in a long spelling session. Depending on the desired size of the training set, this could exceed the reasonable duration of an experiment.

**Figure 2:**
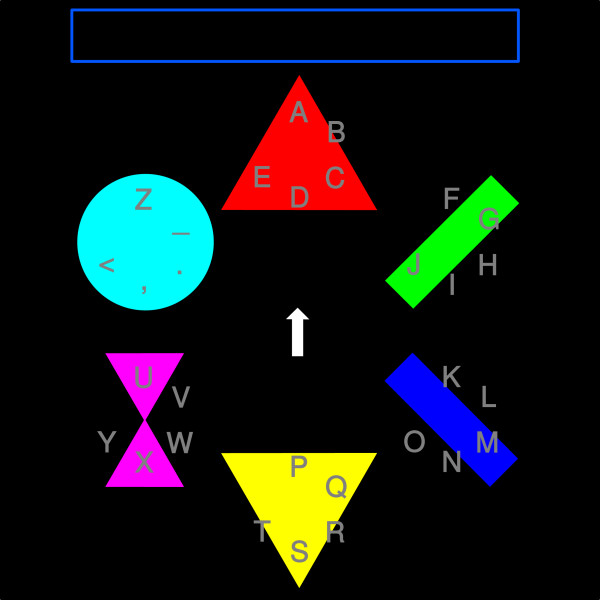
**Calibration Experiment**. The central arrow rotates clockwise and the participant has to press a key when the arrow passes the target symbol. The visual feedback is identical with the feedback of the Center Speller.

As an alternative, a calibration experiment was designed, wherein the participants spell via key press in a much faster way than using a BCI. The spelling process and the appearance of this experiment were designed to be as similar as possible to those of the Center Speller, in order to assure that the classifier trained on this data transfers to the Center Speller application. Just as in the Center Speller, the ErrP Calibration Speller features two levels, one to select the target group, the other one to select the target character. Instead of presenting the elements sequentially in the center of the screen, they are shown in a hexagonal arrangement (Figure 2). A small arrow in the center of the screen is used to select the target. The arrow continuously rotates clockwise with a speed of 240 deg/s and the participant has to press a key when the arrow points on the target symbol. As soon as the key is pressed, feedback identical to the Center Speller feedback is presented: a fixation point is visible for one second, followed by the one second lasting presenting of the selected group or character. By waiting for one second between key press and feedback presentation, muscular influence on the feedback-ERPs is prevented.

In order to obtain error potentials of the type interaction-ErrP, artificial errors were induced. In these cases, the element located on the side opposite to the selected one was selected instead. Choosing the symbol at the opposite side of the screen guaranteed that the participant did not misperceive the interaction error as her/his own error (own errors occurred when the participant hit the key while the arrow was not pointing on the target, *i.e*. too early or too late).

### Procedure

**Figure 3:**
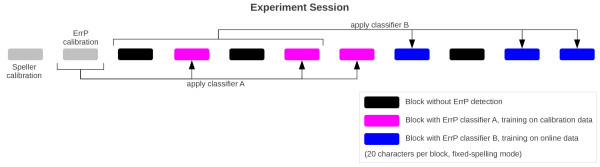
**Session Design**. In total, the experiment consists of 11 blocks. First, the Center Speller is run in offline mode (18 characters), then the ErrP Calibration Speller is run in fixed-spelling mode (83 characters). These two blocks (gray) are used to train the spelling classifier and the ErrP classifier A, respectively. Subsequently, 4 Center Speller blocks are performed in fixed-spelling mode (20 characters per block), and the data is used to train ErrP classifier B. Then the last 5 Center Speller blocks are performed. The three different conditions have been interleaved during the 9 blocks: spelling without ErrP detection (black), spelling with ErrP detection using ErrP classifier A (magenta) and spelling with ErrP detection using ErrP classifier B (blue).

The course of the experimental session is depicted in Figure 3. Prior to the experiment, participants were instructed to relax their muscles and to avoid eye blinks and eye movements during the trials. For the Calibration Speller experiments, participants were asked to place their dominant hand on the keyboard in a relaxed position and press the key with the index finger only. The session started with one block of speller training (Center Speller in offline mode with 10 sequence repetitions). During training, the spelling process was predefined and the participant had to attend the indicated symbols while EEG was recorded for offline analysis. Subsequently, the ErrP Calibration Speller experiment was performed in fixed-spelling mode with 15% artificial errors. In fixed-spelling mode, the participant had to copy a given text and correct all errors that occurred during the spelling process. Based on these two calibration blocks, the spelling classifier and 'ErrP classifier A' were trained. Furthermore, the bias of the ErrP classifier was adjusted to have a false alarm rate of at most 5%. The false alarm rate indicates how many trials were classified as being wrong although the selection was correct, whereas the hit rate indicates the fraction of successfully detected erroneous trials. At the same time, the number of sequence repetitions was adjusted to obtain a spelling accuracy close to, but higher than, 70%. Nine online spelling blocks (Center Speller in fixed-spelling mode) were performed. After completion of the fourth block, another ErrP classifier was trained on the online data, referred to as 'ErrP classifier B'. In other words, there were three spelling conditions during the nine online blocks: spelling without ErrP detection, spelling with ErrP detection using classifier A (trained on the calibration data) and spelling with ErrP detection using classifier B (trained on the online spelling data). The order of the conditions was the same for all participants (as shown in Figure 3).

### Participants

Twelve participants (7 males and 5 females), aged 23-31 years (*μ *= 26), participated in the study. One participant was excluded due to lack of BCI-control. The spelling accuracy for this participant was below 50% which made it impossible to spell and the experiment was aborted. All but one participant were right-handed and all had normal or corrected-to-normal visual acuity. Normal color vision in all but one participant (iac) was confirmed using the Ishihara color vision test [35]. All participants gave written consent and the study was performed in accordance with the Declaration of Helsinki.

### Apparatus

EEG was recorded using a Brain Products (Munich, Germany) actiCAP active electrode system with 64 electrodes and Brain Amp amplifiers sampling at a rate of 1000 Hz. The electrodes were placed according to the international 10-10 system at positions Fp1,2, AF3,4,7,8, Fz, F1-10, FCz, FC1-6, FT7,8, T7,8, Cz, C1-6, TP7,8, CPz, CP1-6, Pz, P1-10, POz, PO3,4,7,8, Oz and O1,2. One electrode was placed under the right eye and labeled as EOGvu. Active electrodes were referenced to a nose electrode, using forehead ground. Impedances were kept below 15 kΩ. EEG signals were hardware filtered at 0.016-250 Hz. The stimuli were presented on a 24" TFT screen with a resolution of 1920 × 1200*px^2 ^*and a refresh rate of 60 Hz. Participants were seated at 70 cm distance from the screen. To correct the EEG markers for the TFT latency, a photo diode (g.TRIGbox; g.tec medical engineering, Graz, Austria) was attached to the lower left corner of the screen for the first six experiments, registering the exact stimulus onset. The median TFT latencies over the six experiments range from 69 ms to 71 ms and for offline analysis, the mean value of 69.8 ms was added to the EEG marker times of all experiments. The Center Speller and the Calibration Speller were implemented in Python http://www.python.org using the open-source-framework Pyff [36] with VisionEgg [37] and Pygame http://www.pygame.org. Remote-controlling of the experiments, online classification as well as offline analysis was done with an inhouse toolbox using MATLAB (The MathWorks, Natick, MA, USA). The Center Speller is freely available in the Pyff repository (see http://bbci.de/pyff).

### Data analysis

For online classification, the EEG data was downsampled to 100 Hz and baseline corrected for the 200 ms prestimulus interval (both, for the speller stimuli and for the feedbacks, which form the ErrP stimuli).

For offline analysis, the data was lowpass filtered using a Chebyshev filter with 42 Hz passband and 49 Hz stopband and then downsampled to 250 Hz. The continuous signal was divided into epochs ranging from -200 ms to 1200 ms relative to the onset of the stimulus and epochs were baseline corrected for the 200 ms prestimulus interval. Artifacts were detected to account for eye blinks, eye movements, muscular activity and malfunctioning hardware. Trials and channels containing such artifacts were rejected for visual ERP analysis, but not for classification purposes. The artifact detection was done using a variance criterion, *i.e*. channels and trials with too low or too high voltage variance were labeled as contaminated by artifacts, as well as using a min-max criterion, *i.e*. all trials in which the difference between maximum and minimum voltage exceeds 75 μV were labeled as contaminated by artifacts. In the ErrP Calibration Speller experiments, all trials containing errors made by the participant were also rejected.

The signed square of the point-biserial correlation coefficient *sgn r^2 ^*was used for ERP analysis as a measure for the discriminability of two classes (target vs. non-target or error vs. non-error) [38].

The performance of the spelling classifier (*i.e*. classification accuracy) is given as the percentage of correctly selected symbols (per level). The performance of the ErrP classifiers is given in terms of the receiver-operating-characteristic (ROC). An ROC curve can be depicted as a plot of false alarms against hits. Since a good classifier allows for a high hit rate at few false alarms, the area under the ROC curve (AUC) is a commonly used quantification of classifier performance. Furthermore, the accuracy of the ErrP detection is split into hits (an error trial classified as error) and false alarms (a correct trial classified as error), or their respective rates for some analyses. The spelling speed is given in terms of the number of characters that were spelled per minute, abbreviated char/min.

### Classification

All classifiers, that is, the spelling classifier (used for detecting the target symbol) and the two ErrP classifiers (used for detecting ErrPs upon presentation of the feedback), used spatio-temporal features for a linear discriminant analysis with shrinkage of the covariance matrix (see *e.g*. [38]). In the spatial domain, all electrodes except for Fp1,2, AF3,4,7,8 and EOGvu were considered (57 channels) for online classification. In the temporal domain, a heuristic method [38] was used, searching for peaks in the *sgn r^2 ^*between targets and non-targets in the 100-700 ms post-stimulus interval (for the spelling), and between errors and non-errors in the 150-900 ms post-feedback interval (for the ErrP detection), respectively. The heuristic method initially determined 5 temporal intervals, but the number of intervals and the exact temporal position of them could be adjusted by the experimenter before the online operation was started. Finally, the voltages of all selected electrodes were averaged within the selected intervals, constituting a feature vector of length d = nIvals nElectrodes (d = 285 in case of nIvals = 5 intervals and nElectrodes = 57 electrodes).

In an offline analysis, the spatial distribution of the class-discriminative information for ErrP detection was investigated by training one classifier individually for each electrode channel [38]. For each channel, four time intervals were chosen automatically by selecting peaks in the *sgn r^2 ^*values. Voltages were then averaged within the respective intervals resulting in four dimensional features. Training and test sets were chosen in the same two ways as in the online experiments, relating to classifier A and to classifier B. We refer to these classifiers as type-A and type-B classifiers.

To investigate whether artifacts from eye blinks or raised eyebrows could explain the classification results, classification was repeated offline for frontal electrodes (Fp2, F9, F10, EOGvu) that were most susceptible to ocular artifacts.

## Results

### Symbol selection

#### ERPs

**Figure 4:**
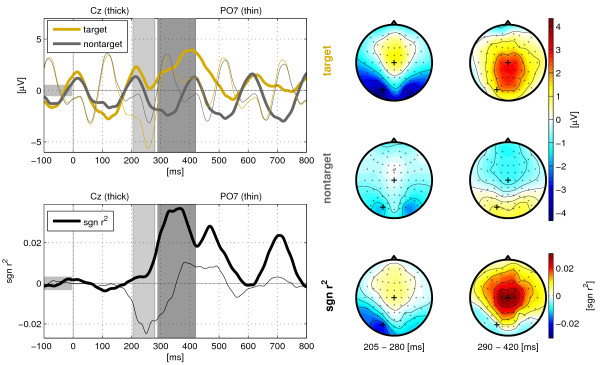
**Event-Related Potentials**. Grand-average of the event-related potentials for target and non-target conditions in the Center Speller experiment. The top plot on the left shows the voltage trace at electrode Cz (thick line) and PO7 (thin line) for the target (orange) and the non-target (dark gray) condition, the bottom plot shows the time evolution of the *sgn r^2 ^*values indicating the target/non-target class difference at those electrodes. The average voltages and *sgn r^2 ^*values of the two intervals (indicated by the gray patches), are shown as scalp topographies on the right side.

Figure 4 shows the grand-average event-related potentials (ERPs) that are related to the presentation of the target and non-target stimuli during the spelling process with the Center Speller. The potentials show an oscillating pattern with a phase length equal to the stimulus onset asynchrony of 200 ms. Due to this short stimulus onset asynchrony, the ERPs of successive presentations overlapped substantially. A negativation at 200-280 ms was present over occipital regions, referred to as N200. This negativation was stronger in the target condition than in the non-target condition and led to a peak in the *sgn r^2 ^*values over left inferotemporal regions. A large positivation in the target condition, the P300 component, was found over central regions 300-500 ms after stimulus onset. The positivation was absent in the non-targets which leaded to high *sgn r^2 ^*values. The P300 reflects the recognition of the rare target event the participant was attending to.

#### Classification

The number of sequence repetitions was set to values between 1 and 7 (*μ *= 2.9, see Table 1). Classification accuracy for levelwise selection (chance level 16.67%) varied significantly between the group level (75.2% ± 3.1) and single character level (82.7% ± 2.4, t = 7.81, *p *< 0.001, see 3^rd ^column of Table 1). The recognition of a single character target is apparently easier than the recognition of a group target consisting of five small characters. The three conditions (spelling without ErrP detection, spelling with ErrP detection with classifier A and B) showed no difference in accuracy of the spelling classifier. A one-way analysis of variance (ANOVA) yielded no significant effect of experimental condition (*p *= .87).

**Table 1:** Results of the online study.

Participant	**Nr**.	Spelling without		Training Set	Performance	Training Set	Performance
code	**Rep**.	ErrP detection		of Classifier A	of Classifier A	of Classifier B	of Classifier B
		**(Accuracy l1, l2/Speed)**		**(Trials/Errors)**	**(AUC/Speed)**	**(Trials/Errors)**	**(AUC/Speed)**

gbo	2	64.7%, 73.7%/0.9		317/47 (14.8%)	0.83/1.5	364/114 (31.3%)	0.97/2.4

bad	2	64.4%, 72.7%/1.2		294/45 (15.3%)	0.85/1.6	481/145 (30.1%)	0.97/2.3

iae	1	66.3%, 75.8%/1.3		309/50 (16.2%)	0.75/2.0	315/62 (19.7%)	0.96/2.8

gbq	2	67.5%, 76.1%/1.2		315/51 (16.2%)	0.62/1.2	402/112 (30.3%)	0.96/2.4

gbt	4	84.1%, 89.1%/2.3		370/59 (15.9%)	0.80/1.8	230/26 (11.3%)	0.93/1.9

iac	2	76.1%, 82.8%/1.7		285/45 (15.8%)	0.83/2.3	291/64 (22.0%)	0.91/2.4

gbn	2	76.8%, 86.6%/1.9		330/51 (15.5%)	0.82/2.4	287/57 (19.9%)	0.87/2.5

gbw	7	81.6%, 85.6%/1.3		415/63 (15.2%)	0.87/1.4	255/35 (13.7%)	0.84/1.3

iau	3	75.3%, 85.8%/1.9		364/60 (16.5%)	0.69/1.6	224/49 (21.9%)	0.79/2.2

mk	3	72.1%, 83.0%/1.9		308/46 (14.9%)	0.69/1.5	290/50 (17.2%)	0.75/1.5

fat	4	98.5%, 99.0%/2.7		379/60 (15.8%)	0.95/2.6	169/2 (1.2%)	0.99/2.7

**Mean**	**2.9**	**75.2%, 82.7%/1.7**		**335/52 (15.6%)**	**0.79/1.8**	**301/66 (19.9%)**	**0.90/2.2**

**SE**	**0.5**	**3.1, 2.4/0.2**		**27/4 (1.3)**	**0.03/0.1**	**27/13 (2.7)**	**0.02/0.1**

Column 2: The number of sequence repetitions used for the Center Speller. Column 3: Overall spelling accuracies for each of the two levels and the spelling speed for spelling without ErrP detection. Columns 4 and 6: The number of training samples and the proportion of error trials for classifier A and B, respectively. Columns 5 and 7: The ErrP detection performance of the two classifiers as area under the ROC curve, as well as the spelling speed in char/min when using the two classifiers, respectively

### ErrP detection

#### ERPs

**Figure 5:**
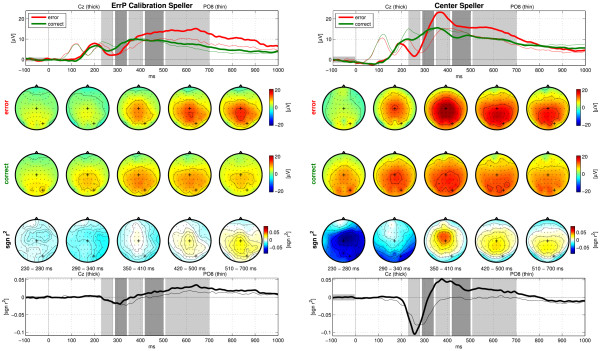
**Error-Related Potentials**. Grand-average of the event-related potentials for error and non-error conditions in the ErrP Calibration Speller (left side) and in the Center Speller (right side) experiment. The top rows show the voltage trace at electrode Cz (thick lines) and PO7 (thin lines) for the error (red) and non-error (green) conditions. The average voltages of five intervals (depicted by the gray patches), are shown as scalp topographies in the two rows below. The third row shows the scalp topographies of the *sgn r^2 ^*values, averaged in these intervals. The bottom plot shows the time evolution of the *sgn r^2 ^*values for electrode Cz and PO7. In the scalp maps, electrodes Cz and PO7 are marked with thick crosses.

The grand-average ERPs with respect to the feedback presentation of error and non-error trials, the error-related potentials, are depicted in Figure 5 for the ErrP Calibration Speller (left) and for the Center Speller experiments (right). In the Center Speller, the responses of both conditions were up to 7 V higher than in the ErrP Calibration Speller. Also, the *sgn r^2 ^*reached higher values in the Center Speller [-0.1, 0.05] than in the ErrP Calibration Speller [-0.025, 0.035]. In both spellers, the error condition (red lines) deviated strongly from the non-error condition (green lines). In the *sgn r^2 ^*values this was reflected by a negativation 200-350 ms after onset of the feedback, referred to as error negativity (*N_E_*), which was not only much stronger in the Center Speller experiments, but also appeared about 40 ms earlier than in the Calibration Speller. The *N_E _*appears first spread across central, temporal and parietal areas, with a peak over central electrode sites and then persisted only over occipital regions with a peak over the right side (*e.g*. electrode PO8). The *N_E _*was followed by a large positivation 350-800 ms after feedback onset, the error positivity (*P_E_*). It appeared first centrally and then moved to centro-parietal regions. Again, the *P_E _*reached much higher values in the Center Speller and it reached its maximum faster (after 380 ms) than in the ErrP Calibration Speller (maximum after 650 ms). The spatio-temporal characteristics of *N_E _*and *P_E _*were in accordance with these found by Combaz *et. al *[31]. The ErrPs observed by Ferrez and Millán [28] looked different, because the EEG signals were processed in different ways. However, by applying a 1-10 Hz bandpass filter as they did and by using the miss-minus-hit curve instead of the *sgn r^2^*, we found similar ErrPs, except for a small time shift (data not shown). This time shift could be due to the monitor latency or the phase shift caused by the bandpass filtering, which might differ in the two settings.

#### Classification

**Figure 6:**
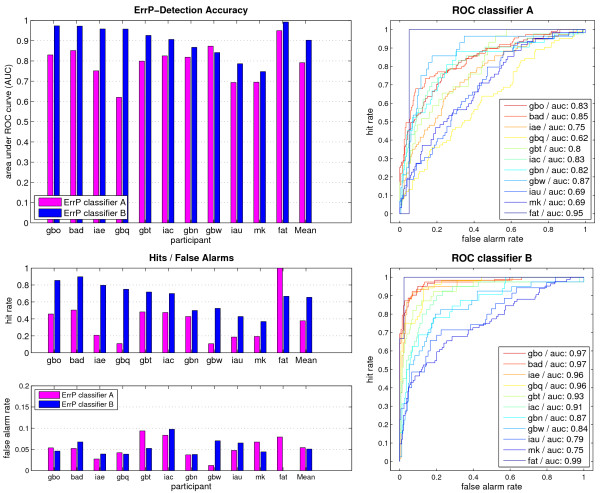
**ErrP Classification**. Performance of the online ErrP classifiers. The AUC for each participant and for the mean are depicted in the top left plot for classifier A (magenta) and classifier B (blue). The hit rates and false alarm rates for each participant and classifier are shown underneath. The right plots show the ROC curves of each participant for classifier A (top) and classifier B (bottom). Classifier B has higher AUCs in all but one participant. The mean AUCs differ more than 10%. This can also be seen in the ROC curves, which reach higher hit rates at lower false alarm rates for classifier B. False alarm rates could be kept around 5% in most participants. In two participants, the false alarm rate approached 10%.

The results of the ErrP classification are summarized in Table 1. The number of training samples for the two classifiers are shown in column 4 and 6, respectively. For classifier A, on average 335 trials, 15.6% of which were errors, were available for training. Classifier B had with 301 trials and 19.9% errors a similar average training set size. For all participants, the performance in terms of the area under the ROC curve was above 0.62 for classifier A and above 0.75 for classifier B. Figure 6 shows the ROC curves for each participant and classifier, as well as the respective AUCs, hit rates and false alarm rates. The performance of classifier B (blue bars) was higher (mean 0.90 AUC) than the performance of classifier A (magenta bars, mean 0.79 AUC) for all but one participant (gbw). A paired-samples t-test on the performance of both classifiers confirms this difference (*t = 3.8, P *< 0.01). The higher performance of classifier B had its origin in the higher hit rates, whereas the false alarm rates were similar for both classifiers (approx. 5%). For participants gbt and iac however, the false alarm rates reached almost 10%, although the classifier was biased in order to keep the false alarm rates below 5%. The ROC curves confirm the advantage of classifier B in reaching higher hit rates at lower false alarm rates in this classifier. The performance of classifier A was not significantly different (*t *= 1.2, *P = *0.25) in the online experiments on the blocks 4, 6 and 7, compared to the performance it achieved in an offline analysis on the blocks 8, 10 and 11 (the blocks where classifier B was used during the online experiments). Thus the advantage of classifier B cannot be explained by an learning effect of the participant.

### Spelling speed improvement

**Figure 7:**
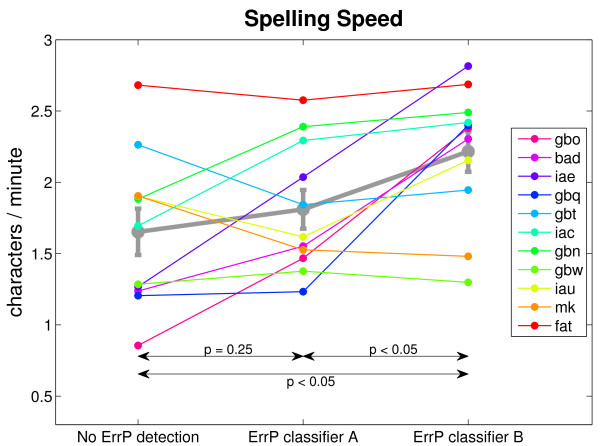
**Spelling Speed**. Spelling Speed in the three conditions: without ErrP detection (left), with ErrP classifier A (middle) and with ErrP classifier B (right). The gray error bars give the mean speed and standard error for each condition. The double arrows indicate the three paired-samples t-tests with the respective *p *values.

Figure 7 compares the spelling speed of the three conditions: Spelling without ErrP detection, spelling with ErrP detection using classifier A and spelling with ErrP detection using classifier B. For most participants the spelling speed increased when using classifier A and increased even more when using classifier B. For two participants (gbw and fat) the speed did not change remarkably. Two other participants (gbt and mk) showed even a reduced speed when using ErrP detection. The average spelling speed was the highest for classifier B with 2.2 char/min. An ANOVA revealed a difference in spelling speed between the three conditions (*F *= 3.89, *p *< 0.05). Tukey-Kramer post-hoc tests showed that spelling speed for classifier B was significantly higher than spelling speed without ErrP detection. The spelling speed obtained with classifier A did not differ significantly from the speed in the other conditions.

**Figure 8:**
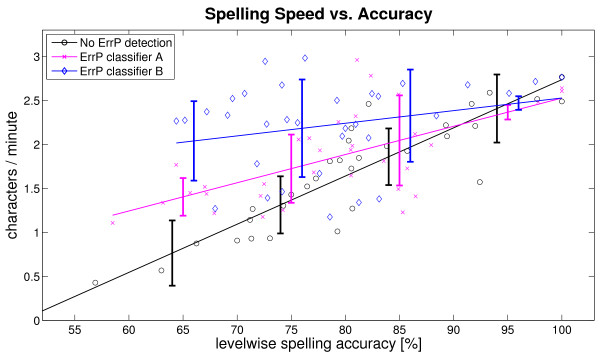
**Speed vs. Accuracy**. Scatter plot of the spelling speed in dependence of the levelwise spelling accuracy. Each data point refers to one experimental block of one participant (nine data points per participant). The blocks in which the ErrP detection was turned off are depicted as black circles, the blocks in which classifier A was used to detect ErrPs are depicted as magenta crosses and the blocks of classifier B are depicted as blue diamonds. For each condition a line was fitted to the data using least squares. The error bars depict the standard deviation of the accuracy in the bands 100-90%, 90-80%, 80-70% and 70-60%.

Figure 8 depicts the relationship between the spelling speed and the spelling accuracy for the three conditions. Not surprisingly, spelling speed was low when spelling accuracy was low, and vice versa. Using ErrP detection, however, the effect of spelling accuracy on speed was attenuated. In case of classifier B (blue line), the mean spelling speed was above 2 char/min even at an spelling accuracy of only 65%, which was more than twice as fast than without ErrP detection (black line). At high accuracies, however, false alarms outweighed the hits, so that fastest spelling was obtained without ErrP detection (2.75 char/min compared to approx. 2.5 char/min for classifiers A and B). Classifier B became advantageous where the black and the blue lines cross, at 95% spelling accuracy. Classifier A (magenta line) became advantageous only below 90% spelling accuracy (crossing of the black and the magenta lines).

### Spatial distribution of discriminative information

**Figure 9:**
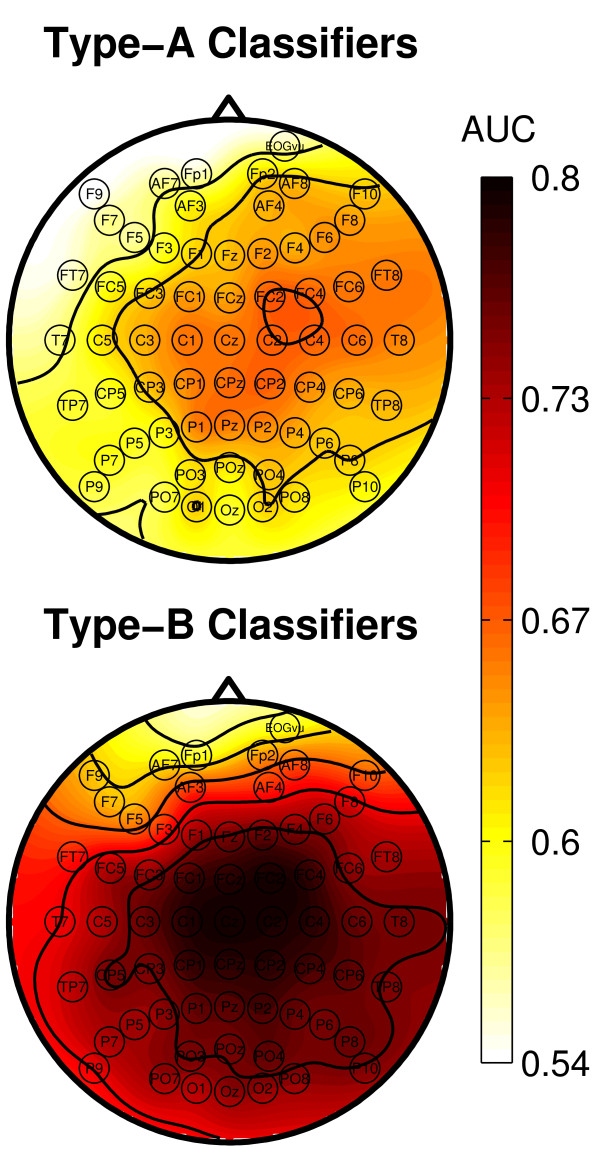
**Spatial Classification**. Spatial distribution of the AUC scores for ErrP classifiers of type-A (top) and type-B (bottom) shown as scalp topography. Classification was performed for each channel separately. Peak performance was reached at central regions for both types (A:0.67, B: 0.8), frontal regions are near chance level. The performance is higher for type-B than for type-A classifiers in all regions.

The spatial distribution of the class-discriminative information for ErrP detection is shown as scalp topographies in Figure 9 (electrode EOGvu, which was placed below the right eye was included in the scalp maps). As in the online experiments, type-A classifiers yielded a lower overall performance compared to type-B classifiers, with peak performance at 0.67 and 0.8, respectively. The performance of the two types of classifiers had a similar spatial distribution as the ErrP components themselves. For type-A classifiers, the highest performance was obtained over central regions, with a bias to the right hemisphere (peak performance at electrodes C2 and FC4). For type-B classifiers, the peak performance was found for electrode Cz and was decreasing towards the periphery.

ErrP detection performance was lower for frontal electrodes (Fp2, F9, F10, EOGvu) than for all other electrodes (type-A: *t *= 2.89, *p *< 0.05; type-B: *t *= 4.42, *p <*0.01). This is in line with Figure 9, where classification performance is at a minimum for frontal channels. These results suggests that ocular artifacts are unlikely to substantially contribute to successful error detection.

## Discussion

Single-trial ErrPs were detected with a mean accuracy of 89.1% (AUC 0.90). The online detection rate was similar to the cross-validation results in offline studies, where 82% [28] and 80% [34] have been reported. ErrP detection using a classifier trained on the online data increased the mean spelling speed by 49.0% compared to the case without ErrP detection. A similar rate of improvement was obtained by [29] with ErrP detection in Matrix Speller experiments (29.5% increase of the bit rate). This illustrates that ERP spellers can be enhanced significantly by detecting and vetoing erroneous decisions of the BCI based on error potentials. Furthermore, the gain in communication speed was relatively higher for participants with a medium or low BCI performance (say, > 10% errors) than for participants with a high BCI performance.

In some cases, ErrP detection could impede spelling speed instead of accelerating it. False alarms prolonged the spelling process because a correct selection was vetoed and had to be repeated. In particular, in cases where participants produced few errors (due to high spelling accuracy), the potential of improvement due to error detection was limited and could easily be outweighed by the detrimental effect of false alarms. This shows that the balance between hits and false alarms of the ErrP classifier has a crucial influence on the overall spelling performance in terms of speed. By moving the decision boundary of the ErrP classifier (ErrP bias), this balance can be controlled by the experimenter. Hence, the trade-off that maximizes communication speed is not only a function of the number of repetitions (which affects the spelling accuracy and thus the speed), but is also affected by the placement of the ErrP bias. Due to the recursive nature of the speller paradigm (the ErrP detector can potentially veto every trial and lead to an infinite loop), finding the optimal trade-off is an intricate problem that will be addressed in future theoretical work. One approach to be mentioned here could be to use estimates of the spelling accuracy for different repetition numbers, together with estimates of the hit rate and false alarm rate of the ErrP classifier for different bias values (both could be obtained *e.g*. from the calibration blocks). Knowing the duration of a Center Speller trial, one could simulate the spelling process for different values of repetition number and ErrP bias in a Monte-Carlo fashion and chose the combination of the parameters that maximizes the speed.

A drawback of using ErrPs is the fact that one has to collect a substantial number of error trials in order to train the ErrP classifier. There are different possible routes to accomplish this. First, one may perform an experiment in two successive stages. In the first stage, spelling would be done without error correction. In the next stage, the trials would have to be labeled and used for training an ErrP classifier (just as we did for classifier B). Second, one could use a calibration phase to collect trials for the ErrP classifier (as we did for classifier A). Regarding the second case, our data show that an ErrP classifier trained in one paradigm (classifier A in the present study) can transfer to a similar paradigm, albeit with a reduced performance. However, the Calibration Speller is not applicable in a clinical context because it involves key presses. However, a calibration experiment that would be completely passive and thus applicable to patients, similar to the offline calibration phase of the spelling classifier, could be used to collect ErrP data for classifier training. It is true that the ErrPs from such a calibration experiment may have large differences to the ErrPs obtained in the Center Speller ('observation ErrPs' instead of 'interaction ErrPs'). Therefore, the applicability of such an approach remains to be investigated.

Ultimately, the utility of ErrP detection is dictated by whether a successful implementation in a clinical setting is feasible. ErrP detection could be relevant to patients because their BCI performance is more variable and often lower than the performance of healthy participants. However, this critically depends on whether error potentials can be detected reliably in patients. Regarding this question, the work of Spüler [29] is instructive. In a clinical study with four patients, ErrPs were classified with an accuracy of 71%. Using a Matrix speller, the bit rate was increased by 35.6% on average. If patients are in a progressed state of the locked-in syndrome, a possible approach for calibration of the ErrP classifier would be to have patients passively observe errors and train on the resulting observation ErrPs, as outlined above [25].

## Conclusion

Concluding, we demonstrated a significant increase of communication speed of gaze-independent ERP spellers when error potentials are detected online. Since BCI performance is often low in patients and successful detection of ErrPs has been demonstrated in ALS patients [29], we believe that ErrP detection can complement conventional BCIs in a clinical application.

## Abbreviations

BCI: brain-computer interface; EEG: electroencephalography; MEG: magnetoencephalography; EOG: electrooculography; ALS: amyotrophic lateral sclerosis; ERP: event-related potential; ErrP: error-related potential; N_E_: error-negativity; P_E_: error-positivity; ROC: receiver-operating-characteristic; AUC: area under the ROC curve; ANOVA: analysis of variance.

## Authors' contributions

NMS, MST and BB conceived and designed the experiments. NMS performed the experiments and analyzed the data. NMS, MST and BB wrote the paper. The authors have declared that no competing interests exist. All authors read and approved the final manuscript.
